# Human Leukocyte Antigen alleles associated with Myalgic Encephalomyelitis/Chronic Fatigue Syndrome (ME/CFS)

**DOI:** 10.1038/s41598-020-62157-x

**Published:** 2020-03-24

**Authors:** Asgeir Lande, Øystein Fluge, Elin B. Strand, Siri T. Flåm, Daysi D. Sosa, Olav Mella, Torstein Egeland, Ola D. Saugstad, Benedicte A. Lie, Marte K. Viken

**Affiliations:** 1 Department of Medical Genetics, University of Oslo and Oslo University Hospital, Oslo, Norway; 2 Institute of Clinical Medicine, University of Oslo, Oslo, Norway; 3 Department of Oncology and Medical Physics, Haukeland University Hospital and Department of Clinical Science, University of Bergen, Bergen, Norway; 4 National Advisory Unit on CFS/ME, Oslo University Hospital, Oslo, Norway; 5 Faculty of Health Science, VID Specialized University, Stavanger, Norway; 6 CFS/ME Center, Oslo University Hospital, Oslo, Norway; 7 Department of Immunology, Oslo University Hospital, Oslo, Norway; 8 Department of Pediatric Research, Oslo University Hospital, University of Oslo, Oslo, Norway

**Keywords:** Genetics research, Risk factors

## Abstract

The etiology and pathogenesis of Myalgic Encephalomyelitis/Chronic Fatigue Syndrome (ME/CFS) are unknown, and autoimmunity is one of many proposed underlying mechanisms. Human Leukocyte Antigen (HLA) associations are hallmarks of autoimmune disease, and have not been thoroughly investigated in a large ME/CFS patient cohort. We performed high resolution *HLA -A*, *-B*, *-C*, *-DRB1*, *-DQB1* and *-DPB1* genotyping by next generation sequencing in 426 adult, Norwegian ME/CFS patients, diagnosed according to the Canadian Consensus Criteria. HLA associations were assessed by comparing to 4511 healthy and ethnically matched controls. Clinical information was collected through questionnaires completed by patients or relatives. We discovered two independent HLA associations, tagged by the alleles HLA-C*07:04 (OR 2.1 [95% CI 1.4–3.1]) and HLA-DQB1*03:03 (OR 1.5 [95% CI 1.1–2.0]). These alleles were carried by 7.7% and 12.7% of ME/CFS patients, respectively. The proportion of individuals carrying one or both of these alleles was 19.2% in the patient group and 12.2% in the control group (OR 1.7 [95% CI 1.3–2.2], p_nc_ = 0.00003). ME/CFS is a complex disease, potentially with a substantial heterogeneity. We report novel HLA associations pointing toward the involvement of the immune system in ME/CFS pathogenesis.

## Introduction

Myalgic Encephalomyelitis/Chronic Fatigue Syndrome (ME/CFS) is a disabling disorder characterized by medically unexplained fatigue, post-exertional malaise and a variety of additional symptoms, such as chronic pain, sleep disturbances and cognitive difficulties. ME/CFS is diagnosed on clinical grounds alone, and different sets of criteria specify the mandatory symptoms as well as recommendations for the exclusion of differential diagnoses^1–3^. The specificity and validity of different diagnostic criteria have been questioned, yet there is no agreement on the level of heterogeneity in ME/CFS, and there is no consensus on how to categorize different subgroups^4–8^.

The pathogenesis and etiology of ME/CFS are unknown, with several models having been proposed^9^. One central hypothesis states that autoimmunity is part of the pathophysiology^10,11^. ME/CFS has been reported to be partly heritable^12,13^, consistent with a multifactorial etiology dependent on both genetic and environmental factors. This is the prevailing model for a vast number of diseases, including established autoimmune diseases (AID). Several publications report immunological alterations among ME/CFS patients, e.g. changes in natural killer (NK) cell function^14,15^, cytokine levels^16,17^, and DNA methylation patterns consistent with immune dysregulation^18^. Some of these findings have failed to reproduce in other studies, which could be due to differences in methodology, the complexity and heterogeneity of ME/CFS, and lack of power due to small sample sizes^19^. Resultingly, the autoimmunity hypothesis warrants further evaluation. A characteristic feature of AID is genetic association with certain human leukocyte antigen (HLA) alleles^20^. Thus, a thorough investigation of HLA associations in ME/CFS is relevant, although HLA associations *per se* cannot be used as evidence regarding disease etiology^21^. Studies of HLA associations in CFS have been published, but with great variation in patient inclusion criteria and HLA typing methodology^22–30^. No reproducible, significant associations are evident across these studies. In the largest study, including 110 patients, the strongest significant association was with HLA-DQ3 with an odds ratio (OR) of 1.8 (95% CI 1.2–2.8)^22^. Associations with HLA alleles DQA1*01, DRB1*13:01 and DQB1*06:02 have also been reported^25,27,29^. The great majority of these studies include less than 50 patients, and are underpowered for the detection of moderate to weak associations. Hence, in this study we aimed to conduct a comprehensive investigation of HLA associations in a large ME/CFS cohort, applying modern, high resolution HLA typing.

## Results

### Characterization of patient and control groups

We included 426 ME/CFS patients and 4511 healthy, ethnically matched controls. All patients had been diagnosed in Norway according to the 2003 Canadian Consensus Criteria^2^, except for four patients where the similarly strict 2010 International Consensus Criteria^3^ were applied. Demographic and clinical characteristics of patients and controls are shown in Table 1. The mean age at diagnosis for ME/CFS patients was 34.7 years, 82.8% were female, and most patients (45.5%) had a disease duration of 5–10 years, from symptom debut to inclusion. 12.5% of the patients had severe or very severe disease (bedridden). An additional 28.6% had moderate to severe disease (strictly housebound). A total of 41.1% of ME/CFS patients were bed- or housebound, and 86.8% of patients were unable to work full or part time the previous 6 months.

**Table 1:** Demographic and clinical characteristics of ME/CFS patients and healthy controls.

	Patients	Controls
Mean age, years (min, max)^a,b^	39.5 (17, 79)	30.6 (19, 52)
Percentage females^a,c^	82.8	59.8
Mean age at diagnosis, years (min, max)^d^	34.7 (8, 65)	—
**Disease duration, percentage of patients**^**e**^		
1–5 years	18.1	—
5–10 years	45.5	—
10–15 years	26.4	—
>15 years	10.1	—
Disease severity, measured by activity assessment in DSQ79*, percentage of patients^**f**^		
Cat.1: Bedridden	12.5	—
Cat.2: Strictly housebound	28.6	—
Cat.3: Light housework	45.7	—
Cat.4: Able to work part time	12.2	—
Cat.5: Able to work full time	0.8	—
Cat.6: Handling some family obligations	0.3	—
Cat.7: Handling work and family obligations	0	—

^a^Valid number of controls: 4510.

Valid number of patients: ^b^426, ^c^424, ^d^345, ^e^288, ^f^385.

*DePaul Symptom Questionnaire, question no. 79^49,50^:

Cat.1: I am not able to work or do anything, and I am bedridden.

Cat.2: I can walk around the house, but I cannot do light housework.

Cat.3: I can do light housework, but I cannot work part-time.

Cat.4: I can only work part time at work or on some family responsibilities.

Cat.5: I can work full time, but I have no energy left for anything else.

Cat.6: I can work full time and finish some family responsibilities but I have no energy left for anything else.

Cat.7: I can do all work or family responsibilities without any problems with my energy.

### HLA alleles associated with ME/CFS

**Figure 1:**
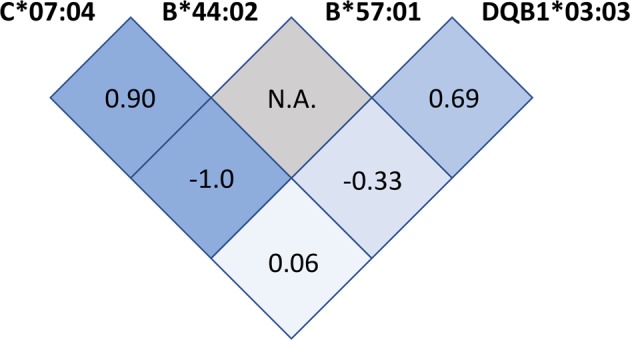
Illustration of Linkage disequilibrium (LD) calculated as D’ values in the patient group between the four alleles with a significant association to ME/CFS. D’ measures the strength of LD between two alleles, and ranges from −1 to 0 for negative LD, and from 0 to 1 for positive LD. The number in each square is the D’ value between the two alleles listed diagonally above the square. Blue colors indicate the strength of LD, with darker colors for stronger LD.

In all patients and controls, we obtained 2nd field resolution genotypes of HLA class I genes *HLA -A, -B* and *-C* and class II genes *HLA -DRB1, -DQB1* and *-DPB1*. This resolution distinguishes HLA alleles that encode specific HLA proteins. No significant deviations from Hardy-Weinberg equilibrium were noted at any HLA loci, neither in the patient group nor in the control group (Supplementary Table S1). Allele frequencies for all observed HLA Class I and Class II alleles are presented in Supplementary Table S2. Global association tests for each HLA locus (Supplementary Table S3) were significant for *HLA-C* (p = 0.04) and *HLA-DQB1* (p = 0.04). When comparing individual allele frequencies between patients and controls, four HLA risk alleles emerged (Table 2): C*07:04 (OR = 2.1 [95% CI 1.4–3.1], p_nc_ = 0.0001, p_c_ = 0.001), B*57:01 (OR = 1.6 [95% CI 1.2–2.3], p_nc_ = 0.004, p_c_ < 0.05), DQB1*03:03 (OR = 1.5 [95% CI 1.1–2.0], p_nc_ = 0.005, p_c_ < 0.05) and B*44:02 (OR = 1.3 [95% CI 1.0–1.6], p_nc_ = 0.03, p_c_ = n.s.). In order to evaluate the dependency of these associations, we measured the degree of linkage disequilibrium (LD) between the four alleles within the patient group (Fig. 1). Strong LD was observed between C*07:04 and B*44:02 (D’ = 0.90) as well as between B*57:01 and DQB1*03:03 (D’ = 0.69), indicating that these alleles may occur on two distinct haplotypes. The first haplotype, C*07:04 - B*44:02, had an estimated frequency of 3.5% in the patient group and 1.7% in the control group, resulting in an OR of 2.1 (95% CI 1.4–3.1, p_nc_ = 0.0002). The second haplotype, B*57:01 - DQB1*03:03, had an estimated frequency of 3.3% in the patient group and 2.0% in the control group, resulting in an OR of 1.7 (95% CI 1.1–2.5 p_nc_ = 0.01). To further evaluate which allele on each of the two haplotypes represents the most significant association, we performed Svejgaard analyses between C*07:04 and B*44:02 as well as between B*57:01 and DQB1*03:03 (Supplementary Data Sheet S4). None of the two alleles on either of the two haplotypes reached significance when testing their independent association, which is not surprising due to the strong LD mentioned above. We report C*07:04 and DQB1*03:03 as tag alleles for the ME/CFS associations, since these alleles occur at the two loci initially showing global association.

**Table 2:** HLA alleles showing association (p_nc_ < 0.05) with ME/CFS in 426 patients and 4511 healthy controls.

HLA allele	ME/CFS, n (%)	Controls, n (%)	OR (95% CI)	p_nc_	p_c_*
C*07:04	33 (3.9)	172 (1.9)	2.1 (1.4–3.1)	0.0001	0.001
B*57:01	39 (4.6)	259 (2.9)	1.6 (1.2–2.3)	0.004	<0.05
DQB1*03:03	56 (6.6)	406 (4.5)	1.5 (1.1–2.0)	0.005	<0.05
B*44:02	105 (12.3)	904 (10.0)	1.3 (1.0–1.6)	0.03	0.3
B*08:01	83 (9.7)	1146 (12.7)	0.7 (0.6–0.9)	0.01	0.1
DPB1*02:01	78 (9.2)	1067 (11.8)	0.7 (0.6–1.0)	0.02	0.1

n = number of alleles.

*Calculated by locus-wise Bonferroni multiple test correction.

We next wanted to make sure that these HLA associations were not due to gender differences between the cases and controls (82.8% vs 59.8% females, respectively). No significant gender differences were observed between the carrier frequencies of these alleles in either cases (Table 3) or controls (C*07:04 had a carrier frequency of 3.7% in females and 4.0% in males; p = 0.6; DQB1*03:03 had a carrier frequency of 9.0% in females and 8.3% in males; p = 0.4). Furthermore, after stratifying cases and controls according to gender, heterogeneity was rejected (p > 0.5) between the OR values obtained for females only and males only, indicating no gender differences between the HLA associations observed in ME/CFS.

**Table 3:** Clinical characteristics of 426 ME/CFS patients: in total and stratified according to the presence of either DQB1*03:03 or C*07:04 or both.

	N (valid)	Total	DQB1* 03:03 +	DQB1* 03:03 -	p_nc_	OR (95% CI)	C* 07:04 +	C* 07:04 -	p_nc_	OR (95% CI)	C*07:04 + and/or DQB1* 03:03 +	C*07:04 - and DQB1* 03:03 -	p_nc_	OR (95% CI)
Gender: Female	424	351 (82.8%)	44 (81.5%)	307 (83.0%)	0.7	0.9 (0.4–1.8)	25 (75.8%)	326 (83.0%)	0.2	0.6 (0.3–1.3)	65 (79.3%)	286 (83.1%)	0.3	0.7 (0.4–1.3)
Symptom start following infectious episode	408	297 (72.8%)	41 (80.4%)	256 (71.7%)	0.2	1.6 (0.8–3.4)	18 (60.0%)	279 (73.8%)	0.1	0.5 (0.2–1.1)	56 (73.7%)	241 (72.6%)	0.8	1.0 (0.6–1.9)
Symptom start following vaccination	407	64 (15.7%)	5 (9.8%)	59 (16.6%)	0.2	0.6 (0.2–1.4)	5 (16.7%)	59 (15.6%)	0.9	1.1 (0.4–3.0)	9 (11.8%)	55 (16.6%)	0.3	0.7 (0.3–1.4)
Previous or current moderate to severe depressive symptoms	407	39 (9.6%)	5 (9.8%)	34 (9.6%)	1.0	1.0 (0.4–2.8)	2 (6.7%)	37 (9.8%)	0.6	0.7 (0.2–2.9)	7 (9.2%)	32 (9.7%)	0.9	0.9 (0.4–2.2)
Comorbidity: Fibromyalgia	408	34 (8.3%)	2 (3.9%)	32 (9.0%)	0.2	0.4 (0.1–1.8)	3 (10.0%)	31 (8.2%)	0.7	1.2 (0.4–4.3)	5 (6.6%)	29 (8.7%)	0.5	0.7 (0.3–2.0)
Comorbidity: Autoimmune disease	405	58 (14.3%)	10 (20.0%)	48 (13.5%)	0.2	1.6 (0.8–3.4)	9 (30.0%)	49 (13.1%)	**0.01**	2.9 (1.2–6.6)	18 (24.0%)	40 (12.1%)	**0.01**	2.3 (1.2–4.3)
1st-degree relative with autoimmune disease	408	154 (37.7%)	24 (47.1%)	130 (36.4%)	0.1	1.5 (0.9–2.8)	7 (23.3%)	147 (38.9%)	0.1	0.5 (0.2–1.1)	30 (39.5%)	124 (37.3%)	0.7	1.1 (0.7–1.8)
1st-degree relative with CFS/ME	403	59 (14.6%)	11 (22.0%)	48 (13.6%)	0.1	1.8 (0.9–3.7)	3 (10.0%)	56 (15.0%)	0.5	0.6 (0.2–2.1)	13 (17.3%)	46 (14.0%)	0.5	1.3 (0.7–2.5)

The two loci *HLA-DRB1* and *HLA-DQB1* are physically close, and exhibit particularly strong LD. In our dataset, DQB1*03:03 occurred most frequently with DRB1*07:01. The haplotype DRB1*07:01 - DQB1*03:03 had estimated frequencies of 4.7% and 2.9% in the patient and control group, respectively (OR 1.7 [95% CI 1.2–2.3], p_nc_ = 0.003). Among patients, this haplotype was without exception estimated to carry DQA1*02:01. Genotype data for the *HLA-DQA1* locus was only obtained for patients, and was not available for the controls.

There were two alleles with a negative association with ME/CFS, suggesting a potential protection, namely B*08:01 (OR = 0.7 [95% CI 0.6–0.9], p_nc_ = 0.01, p_c_ = n.s.) and DPB1*02:01 (OR = 0.7 [95% CI 0.6–0.9], p_nc_ = 0.02, p_c_ = n.s.) (Table 2). These alleles were not in LD (D’ = −0.29), indicating that the associations are independent. The most frequent B*08:01 haplotype in Norway is the highly conserved so-called autoimmune and ancestral AH8.1 haplotype^31^ (C*07:01-B*08:01-DRB1*03:01-DQB1*02:01). This haplotype had reduced estimated frequency in the patient group compared to the control group (8.2% vs. 10.3%, OR = 0.8, p_nc_ = 0.06), albeit not significantly.

### HLA risk allele carriers and clinical characteristics

The proportion of individuals carrying the allele C*07:04 was 7.7% in the patient group and 3.8% in the control group, while 12.7% of the patients and 8.7% of the controls carried DQB1*03:03 (Supplementary Data Sheet S4). The proportion of individuals carrying one or both of the two alleles was 19.2% in the patient group and 12.2% in the control group (OR 1.7, p_nc_ = 0.00003, 95% CI[1.3–2.2]). Table 3 shows the distribution of clinical characteristics in the patient group, including stratification for C*07:04 and/or DQB1*03:03. Neither gender, initiating events, comorbidity of depression or fibromyalgia, nor AID or ME/CFS among 1st degree relatives were associated with the risk alleles. However, ME/CFS patients carrying one or both of the risk alleles had a significantly higher proportion of comorbid AID (OR = 2.3 [95% CI 1.2–4.3], p_nc_ = 0.01). The frequency of comorbid AID was significantly increased also when stratifying for C*07:04 alone (OR = 2.9 [95% CI 1.2–6.6], p_nc_ = 0.01), but not when stratifying for DQB1*03:03 alone (OR = 1.6 [95% CI 0.8–3.4], p_nc_ = n.s.). These patients, carrying HLA risk alleles, had the following AID, ordered by frequency: Hashimoto’s thyreoiditis/hypothyreosis, psoriasis, rheumatoid arthritis, alopecia areata and Crohn’s disease or ulcerative colitis.

## Discussion

In this project, we performed high resolution HLA genotyping by next generation sequencing (NGS) in 426 adult, Norwegian ME/CFS patients, diagnosed according to the Canadian Consensus Criteria^2^. There are no previous publications with comprehensive HLA genotyping by NGS in this patient group. We discovered two independent HLA associations, tagged by the alleles HLA-C*07:04 and HLA-DQB1*03:03.

To our knowledge, associations with *HLA-C* alleles have not previously been studied in ME/CFS. In 1994, Keller *et al*. performed serologic HLA-DR and DQ typing in 110 patients with Chronic fatigue immune dysfunction syndrome (CFIDS)^22^. The patients were diagnosed with the Holmes Criteria^32^, and CFIDS was defined as a subgroup with positive findings in viral reactivation patterns and B- and T-cell tests, indicating post-infectious debut and a certain degree of immune dysfunction. The authors found a significant association (OR = 1.8) with the serotype HLA-DQ3. Serologic HLA typing is of low resolution compared to genetic typing^33^. HLA-DQ3 corresponds to HLA-DQB1*03 in genetic nomenclature, where DQB1*03:03 is one of the three largest subgroups. Higher resolution *HLA-DQB1* typing have been performed in two smaller cohorts (<58 patients), and even though statistically not significant, DQB1*03:03 was observed slightly more frequent among CFS patients, diagnosed with the Fukuda criteria, than among controls^23,25^. Hence, the findings in existing literature is compatible with the association between ME/CFS and DQB1*03:03 in our material.

HLA-B*08:01 showed reduced frequency in ME/CFS compared to controls in our material. This allele most often occur on the haplotype C*07:01-B*08:01-DRB1*03:01-DQB1*02:01, which was also less prevalent among ME/CFS patients in our material. This ancestral haplotype, AH8.1, is a risk factor for a wide variety of AID, including myasthenia gravis, systemic lupus erythematosus and coeliac disease^31^, but protective against rheumatoid arthritis^34,35^. In the existing literature on HLA and CFS, *HLA-DRB1* is the locus most frequently studied. In four out of five studies, the frequency of DR3/DRB1*03 was lower in the patient group^23–25,28^, while in the fifth study the frequency was similar in both groups^22^. Hence, this haplotype seems truly less prevalent among ME/CFS patients.

Some HLA associations previously reported in CFS are not supported by our results^27,29^. The often cited association with DQA1*01 reported by Smith *et al*.^25^ cannot be evaluated in our material since the *HLA-DQA1* locus was not genotyped in the control group. In our patient group, DQA1*01 occurred on haplotype with the following DQB1 alleles, ordered by frequency: DQB1*06:02, DQB1*05:01, DQB1*06:03, DQB1*06:04 and DQB1*05:03, and neither of these (p_nc_ > 0.1, Supplementary Table S2), nor all combined (OR = 1.0, p_nc_ = 0.6) were associated with ME/CFS.

The present HLA study in ME/CFS is to our knowledge the largest performed to date (other studies comprise ≤ 110 CFS patients). Our study had 80% power to discover HLA-associations with OR ≥ 1.5 given an allele frequency > 0.05. Interestingly, both C*07:04 and DQB1*03:03 remained significant after Bonferroni multiple test correction. Notably, we performed locus-wise multiple test correction, i.e. correcting for the number of alleles tested at each locus, since alleles at different HLA loci are in strong LD, and therefore do not represent independent tests. The Bonferroni method is considered a strict multiple test correction^36^, but on the other hand locus-wise correction does not take into account the lack of complete LD between the investigated loci. Taken together, our results need verification in independent cohorts. In general, established HLA associations are reproducible across different populations, but susceptibility loci can also vary between populations^20,21,37,38^. Therefore, HLA associations in ME/CFS should also be investigated in populations of different ancestry.

Both of the ME/CFS associations observed in our data set were evident at 2nd field resolution (i.e. C*07:04 and DQB1*03:03), which distinguishes alleles encoding amino acid differences. Interestingly, the other C*07 and DQB1*03 alleles were not associated, emphasizing the importance of high resolution HLA genotyping.

We report C*07:04 and DQB1*03:03 as tag alleles for two independent HLA risk associations in ME/CFS, as these alleles are in linkage equilibrium (D’ = 0.06). However, they could still be markers for either one common, or two independently associated, variants outside the loci tested in this study. Alternatively, the associated alleles reported herein could constitute a functional relevance themselves. HLA class I alleles, like C*07:04, could influence disease risk through their interactions with CD8 positive cytotoxic T lymphocytes^20,39^. Disturbances in CD8 positive T lymphocytes have been reported in ME/CFS, although the results are somewhat conflicting^10^. Another important function of HLA class I alleles is to serve as ligands for NK cell receptors. Altered numbers and function of NK cells have been reported by several independent researchers in ME/CFS^14,15,40^. The other associated allele, DQB1*03:03, is an HLA class II allele, which is also interesting in regard to the hypothesis of autoimmunity in ME/CFS. In certain well studied AID, associated HLA class II alleles have been shown to exhibit unique peptide binding properties, as well as HLA-TCR restriction, directly influencing the acquired immune response, e.g. with the production of specific auto-antibodies^41^. A dysregulated activity of CD4 positive T lymphocytes, the principal cell type interfering with HLA class II alleles, have been discussed as a central mechanism in ME/CFS^42^. Several studies report increased levels of specific auto-antibodies in ME/CFS patients^43^, e.g. to neurotransmitter receptors, although most of these lack verification in additional cohorts.

Another interesting question is whether these HLA associations are driven by subgroups of patients, and thereby representing stronger risk alleles. This is a relevant aspect in a complex disease like ME/CFS, where different causal mechanisms may be at play in different subgroups. In our study, ME/CFS patients carrying HLA risk alleles had a significantly higher comorbidity of established AID. We are not aware of any publications reporting associations between C*07:04 or DQB1*03:03 and the AID affecting some of our patients. Therefore, it is unlikely that the associations in our material are driven by HLA associations with already established AID. Familial aggregation is observed for many specific AID, as well as for autoimmunity in general^44^. 1st degree relatives of ME/CFS patients in our study have a high prevalence of AID (Table 3). These observations could potentially be due to an element of autoimmunity in ME/CFS, or within a subgroup. No other patient characteristics were dominant among the ME/CFS patients with HLA risk alleles, specifically neither self-reported infectious onset nor current disease severity. It can be argued that the lack of validity of self-reported data precludes the detection of possible subgroup identifiers.

In conclusion, we report novel HLA associations in a large cohort of ME/CFS patients fulfilling the Canadian Consensus Criteria, thereby supporting the involvement of the immune system in the ME/CFS pathogenesis.

## Materials and Methods

This study is approved by the Norwegian Regional Committees for Medical and Health Research Ethics^45^. All methods and data handling were performed according to relevant national and institutional regulations and guidelines. All patients gave informed consent. In three cases, written consent was given by a close relative due to the patient being severely ill and unable to sign. A total of 426 adult, Norwegian ME/CFS patients were included. All had been diagnosed in Norway according to the 2003 Canadian Consensus Criteria^2^, except for four patients where the similarly strict 2010 International Consensus Criteria^3^ were applied. There were three separate recruitment groups for ME/CFS patients: 214 patients were recruited from recent and ongoing trials with Rituximab^46,47^ and Cyclophosphamide (Rekeland IG *et al*., submitted, NCT02444091); 116 patients were recruited from the CFS/ME biobank at Oslo University Hospital; 96 patients were recruited via announcements in patient networks, including patient organizations. Patients from the latter two groups were not included in clinical trials. Duplicates within or between the three recruitment groups were excluded. All patients provided the identity of any 1st, 2nd or 3rd degree relatives with ME/CFS, and we excluded close relatives to ensure that only one patient per extended family was included. Norwegian ethnicity was ensured by evaluation of sur- and family names of all patients, country of birth of parents and grandparents as well as self-perceived ethnicity. The control group consisted of 4511 ethnically matched, healthy individuals drawn from the Norwegian Bone Marrow Donor Registry^48^. Clinical information was collected for the ME/CFS patients through questionnaires completed by patients or close relatives. The categories applied in this study were gender, age at diagnosis, initiating events, disease duration and severity, comorbidities and family history. Most of the questions were based on the DePaul Symptom Questionnaire^49,50^. Infection or vaccination as initiating event was self-reported, and in many cases, the time from event to symptom debut was not specified. The disease severity was assessed with self-reported activity level during the previous 6 months, as stated through the DePaul Symptom Questionnaire, question no. 79.

### HLA genotyping by next generation sequencing

In 426 ME/CFS patients, we performed high resolution, targeted, next generation sequencing (NGS) of HLA class I genes *HLA -A, -B* and *-C* and class II genes *HLA -DRB1, -DQB1, -DPB1, -DQA1*. Amplification and library preparation were performed with kits from GenDx (Utrecht, The Netherlands) and Illumina (San Diego, USA), 2 ×150 bp paired-end sequencing was performed by The Norwegian Sequencing Centre with Illumina MiSeq Reagent Kit v2 (300-cycles), and HLA genotypes were obtained by analyzing sequencing reads with NGSengine from GenDx, using the IMGT/HLA Database^51^. The median sequencing depth was above 150 reads per called base. The 4511 healthy Norwegian controls had previously been HLA typed by NGS^52^. Both patient and control genotypes were analyzed at 2nd field resolution for *HLA -A*, *-B*, *-C*, *-DRB1*, *-DQB1* and *-DPB1*. HLA alleles can be genotyped at resolution level from 1st field to 4th field. 2nd field resolution distinguishes alleles that encode amino acid differences, i.e. specific HLA proteins, and is therefore of great biological relevance. The genotyping success exceeded 99% in the patient group and 99.9% in the control group for all loci. In the control group, alleles were originally identified at a G group resolution, and certain alleles from the patient group were therefore converted to avoid typing method bias (Supplementary Table S5).

Data analyses were performed in Unphased 3.0.10 and Pypop 0.7.0^53,54^. Assessment of Hardy-Weinberg equilibrium was performed with a chi-square test with a significance level of 0.05. Haplotype frequencies were estimated with an expectation-maximization method for unknown gametic phase. Global associations for each locus were calculated with a likelihood ratio test, with a rare allele frequency threshold of 0.01. Genetic associations were investigated on allelic and haplotypic levels, and ORs with 95% confidence intervals (95% CI) were calculated with Woolf’s formula comprising Haldane’s correction. Risk allele ORs were calculated also with gender stratification, and homogeneity tests were performed with the logit-based estimator. LD calculations and Svejgaard tests were carried out to examine the degree of independence between the associated alleles^55^. The LD measure D’ was calculated according to the formula D’ = D/D_max_, where D_max_ = min [p_A_(1-p_B_), (1-p_A_)p_B_] for D > 0, D_max_ = min [p_A_p_B_, (1-p_A_)(1-p_B_)] for D < 0, D is the standard mathematical definition of LD between alleles A and B^56^, and p is the frequency of the stated allele. Because of the comparison of multiple allele frequencies we performed locus-wise Bonferroni correction. For each locus, non-corrected p-values were multiplied by the total number of alleles detected at that locus, excluding alleles with a frequency less than 3% in both the control group and the patient group. The significance level was 0.05. Only haplotypes consisting of associated alleles were investigated, and multiple test correction was therefore not applied on the haplotypic level.

### Investigation of clinical data

The clinical information was gathered separately for each of the three recruitment groups, controlled in one common database, and exported to SPSS^57^ for statistical analyses. The patient group was stratified according to the presence of specific HLA alleles, and eight dichotomized clinical variables were assessed with OR calculation by binary logistic regression, and chi-square significance testing.

### Restrictions on the availability of material

Individual genotypes of patients are not made available due to Norwegian privacy regulations and laws.

## Supplementary information

Supplementary table 1.

Supplementary table 2.

Supplementary table 3.

Supplementary dataset 4.

Supplementary table 5.

Supplementary tables 1, 2, 3 and 5.
