# Supplementary material for: Human Leukocyte Antigen alleles associated with Myalgic Encephalomyelitis/Chronic Fatigue Syndrome (ME/CFS)
Source: Sci Rep. 2020 Mar 24;10:5267. doi: 10.1038/s41598-020-62157-x (PMC7093502; doi:10.1038/s41598-020-62157-x)
Supplement: Supplementary file 6 — Supplementary tables 1, 2, 3 and 5. [file 41598_2020_62157_MOESM6_ESM.pdf]

Supplementary information for:
"Human Leukocyte Antigen alleles associated with Myalgic Encephalomyelitis/Chronic Fatigue Syndrome (ME/CFS)".

Authors: Asgeir Lande*, Øystein Fluge, Elin B. Strand, Siri T. Flåm, Daysi D. Sosa, Olav Mella, Torstein Egeland, Ola D. Saugstad, Benedicte A. Lie, Marte K. Viken.
*Correspondence: Asgeir Lande, asgeir.lande@medisin.uio.no

Please note: Supplementary Data Sheet S4 is not included in this PDF-file as we found it more suitable to upload it as a separate excel-sheet.

Table S1
Hardy-Weinberg Equilibrium parameters for 6 HLA loci at 2nd field resolution among a) 426 ME/CFS patients, and b) 4511 healthy controls

a)

Locus	Obs Het	Exp Het	P-HWE
**HLA-A**	346	349.9	0.83
**HLA-C**	390	381.2	0.65
**HLA-B**	393	389.6	0.86
**HLA-DRB1**	379	382.4	0.86
**HLA-DQB1**	377	375.0	0.92
**HLA-DPB1**	317	312.0	0.78

b)

Locus	Obs Het	Exp Het	P-HWE
**HLA-A**	3724	3751.7	0.65
**HLA-C**	4043	4034.0	0.89
**HLA-B**	4114	4126.8	0.84
**HLA-DRB1**	4121	4096.9	0.71
**HLA-DQB1**	3966	3936.7	0.64
**HLA-DPB1**	3394	3409.9	0.79

Obs Het: Observed Heterozygosity; Exp Het: Expected Heterozygosity; P-HWE: p-value for Hardy-Weinberg Equilibrium deviation.

Table S2

Allele count and frequency for all detected HLA alleles among patients and controls. HLA Class I and Class II loci are listed in separate tabs

		ME/CFS patients		Controls	
Locus	Allele	Allele count	Allele frequency	Allele count	Allele frequency
HLA-A	01:01	117	0.1373	1433	0.1588
	01:02	0	0.0000	2	0.0002
	02:01	293	0.3439	2889	0.3202
	02:02	0	0.0000	3	0.0003
	02:05	6	0.0070	61	0.0068
	02:06	5	0.0059	12	0.0013
	02:07	0	0.0000	4	0.0004
	02:08	0	0.0000	1	0.0001
	02:17	0	0.0000	1	0.0001
	02:20	0	0.0000	1	0.0001
	02:118	0	0.0000	1	0.0001
	02:474	0	0.0000	1	0.0001
	02:581	0	0.0000	2	0.0002
	03:01	137	0.1608	1401	0.1553
	03:02	0	0.0000	7	0.0008
	03:17	0	0.0000	1	0.0001
	03:56	0	0.0000	1	0.0001
	03:62	0	0.0000	1	0.0001
	03:201	0	0.0000	1	0.0001
	11:01	46	0.0540	547	0.0606
	23:01	12	0.0141	121	0.0134
	23:06	0	0.0000	1	0.0001
	24:02	74	0.0869	772	0.0856
	24:03	4	0.0047	13	0.0014
	24:07	0	0.0000	3	0.0003
	25:01	26	0.0305	222	0.0246
	25:11	0	0.0000	1	0.0001
	26:01	14	0.0164	147	0.0163
	26:08	2	0.0024	10	0.0011
	29:01	4	0.0047	7	0.0008
	29:02	21	0.0247	181	0.0201
	30:01	2	0.0024	56	0.0062
	30:02	4	0.0047	27	0.0030
	30:04	0	0.0000	9	0.0010
	31:01	24	0.0282	301	0.0334
	31:08	0	0.0000	1	0.0001
	32:01	18	0.0211	300	0.0333
	32:63	0	0.0000	1	0.0001
	33:01	1	0.0012	31	0.0034
	33:03	1	0.0012	19	0.0021
	34:01	0	0.0000	2	0.0002
	34:02	1	0.0012	1	0.0001

	66:01	2	0.0024	30	0.0033
	68:01	35	0.0411	365	0.0405
	68:02	2	0.0024	22	0.0024
	68:35	0	0.0000	1	0.0001
	69:01	1	0.0012	4	0.0004
	74:01	0	0.0000	1	0.0001
	74:03	0	0.0000	2	0.0002
	74:05	0	0.0000	1	0.0001
	Total	852	0.99998	9022	0.9996
HLA-C	01:02	31	0.0364	393	0.0436
	02:02	36	0.0423	457	0.0507
	02:10	0	0.0000	4	0.0004
	03:02	2	0.0024	18	0.0020
	03:03	50	0.0587	559	0.0620
	03:04	126	0.1479	1380	0.1530
	03:05	0	0.0000	2	0.0002
	03:14	2	0.0024	3	0.0003
	03:220	0	0.0000	2	0.0002
	04:01	80	0.0939	782	0.0867
	04:03	0	0.0000	2	0.0002
	04:15	0	0.0000	2	0.0002
	04:09N	1	0.0012	0	0.0000
	05:01	88	0.1033	792	0.0878
	05:09	0	0.0000	1	0.0001
	06:02	56	0.0657	549	0.0609
	06:26	0	0.0000	1	0.0001
	07:01	111	0.1303	1383	0.1533
	07:02	154	0.1808	1540	0.1707
	07:04	33	0.0387	172	0.0191
	07:21	0	0.0000	4	0.0004
	07:24	1	0.0012	3	0.0003
	07:36	0	0.0000	1	0.0001
	07:51	0	0.0000	3	0.0003
	07:124	0	0.0000	1	0.0001
	07:212	0	0.0000	1	0.0001
	07:XX	0	0.0000	1	0.0001
	08:01	2	0.0024	19	0.0021
	08:02	12	0.0141	193	0.0214
	12:02	0	0.0000	20	0.0022
	12:03	25	0.0293	265	0.0294
	14:02	3	0.0035	67	0.0074
	15:02	16	0.0188	147	0.0163
	15:04	0	0.0000	5	0.0006
	15:05	1	0.0012	5	0.0006
	15:06	0	0.0000	2	0.0002
	15:11	0	0.0000	3	0.0003
	16:01	17	0.0200	193	0.0214

	16:02	3	0.0035	8	0.0009
	16:04	0	0.0000	1	0.0001
	17:01	2	0.0024	35	0.0039
	18:01	0	0.0000	3	0.0003
	Total	852	0.99999	9022	1.0000
HLA-B	07:02	138	0.1620	1433	0.1588
	07:04	2	0.0024	3	0.0003
	07:05	1	0.0012	5	0.0006
	07:31	0	0.0000	1	0.0001
	08:01	83	0.0974	1146	0.1270
	08:04	1	0.0012	4	0.0004
	08:26	0	0.0000	1	0.0001
	13:01	0	0.0000	1	0.0001
	13:02	6	0.0070	95	0.0105
	14:01	7	0.0082	94	0.0104
	14:02	6	0.0070	102	0.0113
	15:01	75	0.0880	908	0.1006
	15:02	0	0.0000	3	0.0003
	15:03	1	0.0012	5	0.0006
	15:07	2	0.0024	15	0.0017
	15:13	0	0.0000	1	0.0001
	15:16	0	0.0000	4	0.0004
	15:17	1	0.0012	5	0.0006
	15:18	1	0.0012	9	0.0010
	15:21	0	0.0000	1	0.0001
	15:24	0	0.0000	1	0.0001
	15:25	0	0.0000	1	0.0001
	15:39	0	0.0000	1	0.0001
	15:110	0	0.0000	1	0.0001
	15:363	0	0.0000	1	0.0001
	18:01	34	0.0399	330	0.0366
	18:03	0	0.0000	1	0.0001
	27:02	1	0.0012	18	0.0020
	27:05	42	0.0493	525	0.0582
	35:01	53	0.0622	543	0.0602
	35:02	1	0.0012	11	0.0012
	35:03	7	0.0082	70	0.0078
	35:08	3	0.0035	13	0.0014
	35:10	0	0.0000	1	0.0001
	35:17	0	0.0000	1	0.0001
	35:XX	0	0.0000	1	0.0001
	37:01	9	0.0106	122	0.0135
	38:01	5	0.0059	50	0.0055
	39:01	4	0.0047	55	0.0061
	39:05	0	0.0000	2	0.0002
	39:06	7	0.0082	65	0.0072
	39:24	0	0.0000	1	0.0001

40:01	97	0.1139	940	0.1042
40:02	11	0.0129	161	0.0179
40:06	0	0.0000	3	0.0003
40:94	0	0.0000	1	0.0001
41:01	0	0.0000	9	0.0010
41:02	1	0.0012	23	0.0026
42:02	0	0.0000	3	0.0003
44:02	105	0.1232	904	0.1002
44:03	27	0.0317	289	0.0320
44:04	0	0.0000	1	0.0001
44:05	1	0.0012	8	0.0009
44:08	0	0.0000	1	0.0001
44:XX	0	0.0000	1	0.0001
45:01	6	0.0070	41	0.0045
46:01	0	0.0000	5	0.0006
47:01	3	0.0035	24	0.0027
48:01	2	0.0024	13	0.0014
49:01	9	0.0106	110	0.0122
50:01	3	0.0035	29	0.0032
50:02	0	0.0000	4	0.0004
51:01	36	0.0423	309	0.0343
51:05	0	0.0000	2	0.0002
51:08	1	0.0012	3	0.0003
51:09	0	0.0000	1	0.0001
52:01	0	0.0000	22	0.0024
53:01	1	0.0012	12	0.0013
54:01	0	0.0000	2	0.0002
55:01	12	0.0141	105	0.0116
55:02	0	0.0000	1	0.0001
56:01	3	0.0035	47	0.0052
57:01	39	0.0458	259	0.0287
57:02	0	0.0000	4	0.0004
58:01	5	0.0059	31	0.0034
58:02	0	0.0000	1	0.0001
73:01	0	0.0000	3	0.0003
Total	852	0.99996	9022	0.9993

		ME/CFS patients		Controls	
Locus	Allele	Allele count	Allele frequency	Allele count	Allele frequency
HLA-DRB1	01:01	77	0.0912	761	0.0844
	01:02	0	0.0000	41	0.0045
	01:03	6	0.0071	123	0.0136
	03:01	96	0.1137	1231	0.1365
	03:14	1	0.0012	0	0.0000
	04:01	101	0.1197	1035	0.1148
	04:02	0	0.0000	18	0.0020
	04:03	5	0.0059	73	0.0081

	04:04	42	0.0498	537	0.0596
	04:05	1	0.0012	30	0.0033
	04:06	1	0.0012	4	0.0004
	04:07	10	0.0119	68	0.0075
	04:08	3	0.0036	36	0.0040
	04:10	0	0.0000	1	0.0001
	04:38	0	0.0000	1	0.0001
	07:01	86	0.1019	776	0.0861
	08:01	36	0.0427	366	0.0406
	08:02	0	0.0000	14	0.0016
	08:03	0	0.0000	16	0.0018
	08:04	3	0.0036	4	0.0004
	08:10	0	0.0000	1	0.0001
	09:01	14	0.0166	142	0.0158
	10:01	4	0.0047	68	0.0075
	11:01	28	0.0332	310	0.0344
	11:02	0	0.0000	14	0.0016
	11:03	2	0.0024	35	0.0039
	11:04	7	0.0083	59	0.0065
	11:14	1	0.0012	5	0.0006
	11:28	0	0.0000	1	0.0001
	12:01	23	0.0273	195	0.0216
	12:02	0	0.0000	3	0.0003
	13:01	64	0.0758	703	0.0780
	13:02	60	0.0711	527	0.0584
	13:03	1	0.0012	30	0.0033
	13:04	0	0.0000	1	0.0001
	13:06	0	0.0000	1	0.0001
	13:15	0	0.0000	1	0.0001
	14:01	24	0.0284	226	0.0251
	14:02	2	0.0024	11	0.0012
	14:03	0	0.0000	1	0.0001
	14:04	0	0.0000	3	0.0003
	14:05	0	0.0000	1	0.0001
	14:07	0	0.0000	1	0.0001
	14:22	0	0.0000	1	0.0001
	15:01	145	0.1718	1491	0.1653
	15:02	0	0.0000	17	0.0019
	16:01	1	0.0012	32	0.0035
	16:02	0	0.0000	3	0.0003
	Total	844	1.0000	9018	1.0000

HLA-DQB1	02:01	142	0.1667	1744	0.1934
	02:14	0	0.0000	1	0.0001
	03:01	128	0.1502	1215	0.1347
	03:02	102	0.1197	1262	0.1399
	03:03	56	0.0657	406	0.0450
	03:04	1	0.0012	15	0.0017

	03:05	0	0.0000	3	0.0003
	04:01	0	0.0000	1	0.0001
	04:02	41	0.0481	391	0.0434
	05:01	82	0.0962	965	0.1070
	05:02	1	0.0012	42	0.0047
	05:03	24	0.0282	227	0.0252
	05:04	5	0.0059	8	0.0009
	06:01	0	0.0000	21	0.0023
	06:02	142	0.1667	1472	0.1632
	06:03	67	0.0786	725	0.0804
	06:04	56	0.0657	462	0.0512
	06:09	4	0.0047	55	0.0061
	06:16	0	0.0000	2	0.0002
	06:164	0	0.0000	1	0.0001
	06:XX	1	0.0012	0	0.0000
	Total	852	1.0000	9018	0.9999

HLA-DPB1	01:01	43	0.0506	611	0.0678
	02:01	78	0.0918	1067	0.1183
	02:02	7	0.0082	39	0.0043
	03:01	108	0.1271	1003	0.1112
	04:01	402	0.4729	4010	0.4447
	04:02	97	0.1141	1033	0.1145
	05:01	28	0.0329	243	0.0270
	06:01	8	0.0094	144	0.0160
	09:01	3	0.0035	46	0.0051
	10:01	6	0.0071	104	0.0115
	11:01	15	0.0177	147	0.0163
	13:01	10	0.0118	99	0.0110
	14:01	9	0.0106	84	0.0093
	15:01	6	0.0071	52	0.0058
	16:01	3	0.0035	85	0.0094
	17:01	6	0.0071	60	0.0067
	19:01	8	0.0094	95	0.0105
	20:01	7	0.0082	52	0.0058
	23:01	4	0.0047	34	0.0038
	24:01	0	0.0000	2	0.0002
	26:01	0	0.0000	2	0.0002
	31:01	0	0.0000	1	0.0001
	35:01	0	0.0000	1	0.0001
	45:01	1	0.0012	0	0.0000
	51:01	0	0.0000	1	0.0001
	71:01	1	0.0012	0	0.0000
	115:01	0	0.0000	1	0.0001
	129:01	0	0.0000	1	0.0001
	424:01	0	0.0000	1	0.0001
	Total	850	1.0000	9018	0.9999

Table S3

Tests of global association

Locus	Likelihood ratio chisq	No. of alleles	df	p-value
HLA-A	11.01	12	11	0.443
HLA-C	22.86	14	13	0.043
HLA-B	26.21	18	17	0.071
HLA-DRB1	15.08	15	14	0.373
HLA-DQB1	17.69	10	9	0.039
HLA-DPB1	17.2	12	11	0.102

df: Degrees of freedom

Table S5

Conversion of alleles in the patient group (N = 426) belonging to G groups differing on 2nd field resolution

**Locus**	**Allele**	**No. of alleles**	**Belonging to G group**	**Used in analysis**
HLA-C	01:127	6	01:02:01G	01:02
HLA-C	07:18	2	07:01:01G	07:01
HLA-C	17:03	2	17:01:01G	17:01
HLA-B	15:220	1	15:03:01G	15:03
HLA-B	44:27	1	44:02:01G	44:02
HLA-DRB1	01:77	1	01:01:01G	01:01
HLA-DRB1	14:54	24	14:01:01G	14:01
HLA-DQB1	02:02	49	02:01:01G	02:01
HLA-DQB1	03:19	1	03:01:01G	03:01
HLA-DPB1	104:01	3	03:01:01G	03:01
HLA-DPB1	463:01	1	04:02:01G	04:02
